# Functions and mechanisms of circular RNAs in cancer radiotherapy and chemotherapy resistance

**DOI:** 10.1186/s12943-020-01180-y

**Published:** 2020-03-14

**Authors:** Chaochu Cui, Jianbo Yang, Xiao Li, Dongling Liu, Liwu Fu, Xianwei Wang

**Affiliations:** 1 Henan Key Laboratory of Medical Tissue Regeneration, College of Basic Medical Sciences, Xinxiang Medical University, Xinxiang, Henan China; 2 School of Laboratory Medicine, Xinxiang Medical University, Xinxiang, Henan China; 3 State Key Laboratory of Oncology in South China; Collaborative Innovation Center for Cancer Medicine, Sun Yat-sen University Cancer Center, Guangzhou, China

**Keywords:** circRNAs, Cancer therapy, Sensitivity, Radioresistance, Chemoresistance, Multidrug resistance

## Abstract

Circular RNAs (circRNAs), one type of non-coding RNA, were initially misinterpreted as nonfunctional products of pre-mRNA mis-splicing. Currently, circRNAs have been proven to manipulate the functions of diverse molecules, including non-coding RNAs, mRNAs, DNAs and proteins, to regulate cell activities in physiology and pathology. Accumulating evidence indicates that circRNAs play critical roles in tumor genesis, development, and sensitivity to radiation and chemotherapy. Radiotherapy and chemotherapy are two primary types of intervention for most cancers, but their therapeutic efficacies are usually retarded by intrinsic and acquired resistance. Thus, it is urgent to develop new strategies to improve therapeutic responses. To achieve this, clarification of the underlying mechanisms affecting therapeutic responses in cancer is needed. This review summarizes recent progress and mechanisms of circRNAs in cancer resistance to radiation and chemotherapy, and it discusses the limitations of available knowledge and potential future directions.

## Introduction

Circular RNAs (circRNAs), a type of non-coding RNA (ncRNA), were first reported nearly 4 decades ago. They were initially disregarded as functionless products of pre-mRNA splicing errors [1, 2]. Since circRNAs have been identified to have a comparable level to their canonical linear counterparts, they have been suggested as pervasive regulatory molecules [3, 4].

To date, many circRNAs have been identified and proven to be vital in many diseases, such as cancer [5]. CircRNAs regulate tumor genesis, development, proliferation, migration, invasion and sensitivity to therapy [6]. The investigations of long ncRNAs (lncRNAs) and microRNAs (miRNAs), another two types of ncRNAs, have been extensively reported. However, studies of circRNAs in cancer, especially in cancer resistance to radiation and chemotherapy, are still at the nascent stage [7–10].

Inducing tumor cell death is one of the primary effects of radiotherapy and chemotherapy. However, escaping from death, one of the typical characteristics of cancer cells, leads to resistance to therapy, recurrence and poor prognosis of cancers [11]. The mechanisms underlying escape from death include elevated functions of drug efflux pumps, cell stemness, enhanced phagocytosis, and so on [12–16]. It has been increasingly demonstrated that circRNAs manipulate the abovementioned events. This review summarizes the recent progress in the understanding of the mechanisms of cancer resistance potentially related to circRNAs and the functions of circRNAs in radiotherapy and chemotherapy resistance. It also discussed the limitations of available knowledge and future potential directions.

## Biogenesis and general functions of circRNAs

**Fig. 1:**
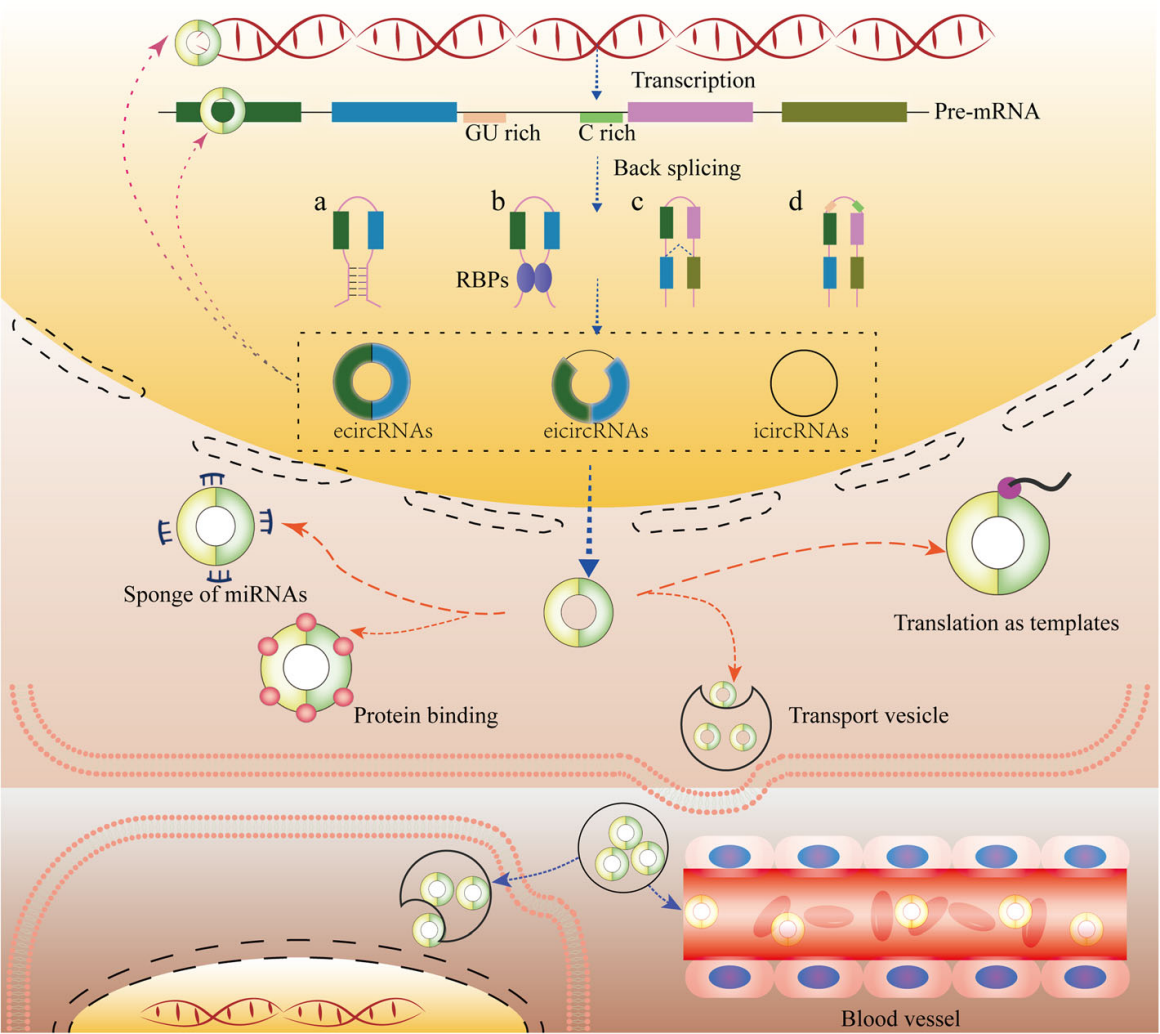
Biogenesis, distribution and function of circRNAs. CircRNAs are generated from back splicing of pre-mRNAs in different manners (a, b, c and d). CircRNAs can regulate the activities and functions of DNAs, RNAs and proteins in host cells. In addition, they can be secreted into the extracellular space and transported into adjacent cells or body fluids to regulate cell activities. a. Base pairing-dependent circularization; b. RNA-binding protein (RBP)-dependent circularization; c. Lariat-driven circularization; d. GU-rich and C-rich element-dependent circularization

The length of conserved circRNAs ranges from hundreds to thousands of nucleotides. They are usually generated from nonsequential back-splicing of pre-mRNA transcripts or back fusion of linear RNAs. CircRNAs with 3′ and 5′ ends covalently joined to form circular loop structures without free ends are insusceptible to RNase R and exonucleolytic degradation. This grants them higher stability than linear transcripts [3, 4]. The intergenic circRNAs can be loosely classified into three types (Fig. 1): exonic circRNAs (ecircRNAs), which only contain exons and represent the majority of circRNAs; intronic circRNAs (icircRNAs), which only contain introns; and exon-intron circRNAs (eicircRNAs), which contain both exons and introns [17].

CircRNAs were discovered serendipitously in the early 1970s and disregarded for a long time [1, 2]. Currently, a growing number of circRNAs have been purposely identified with advances in high-throughput sequencing technologies and investigated by bioinformatics methods [18–20]. The classic and major function of circRNAs is serving as molecular sponges of specific miRNAs to regulate mRNA stability and translation [21, 22]. In addition, circRNAs also act as sponges of proteins, as enhancers or coordinators of proteins, mRNAs and DNAs, and as templates for translation (Fig. 1) [17, 23–27].

## Actions of circRNAs in cancer

CircRNAs are promising cancer biomarkers for clinical diagnosis and prognosis because of their high stability and abundance in body fluids (Fig. 1a). The effective circRNAs can be loosely divided into two groups depending on their functions in cancer: suppressors (inhibitors of resistance) and promoters (enhancers of resistance) [6, 28–30]. For instance, circRHOT1 is upregulated in hepatocellular carcinoma (HCC), while circRNA_101505 is downregulated in cisplatin-resistant HCC tissues, and both are related to the survival of HCC patients [23, 31].

## Mechanisms mediating radioresistance and chemoresistance potentially related to circRNAs in cancer

**Fig. 2:**
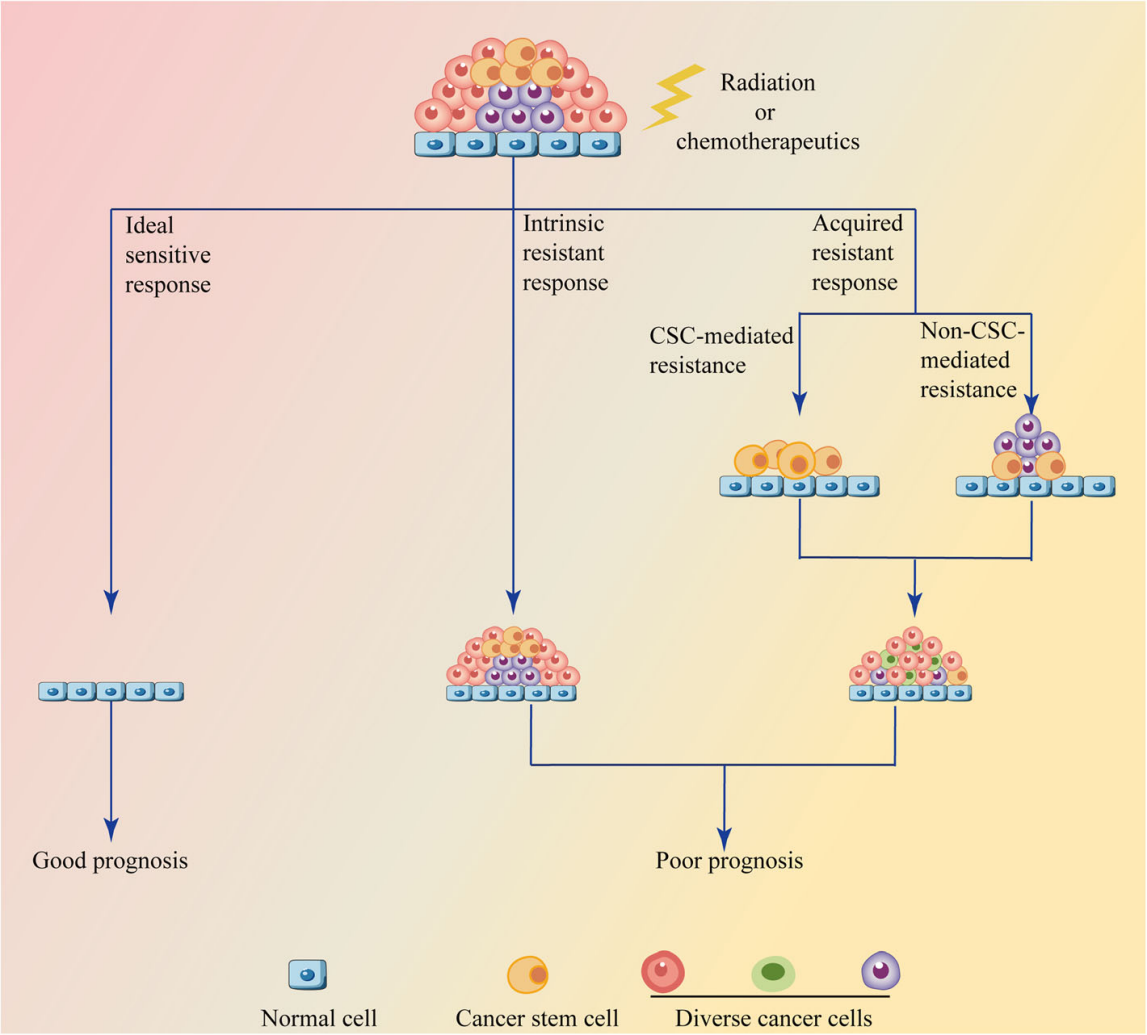
Responses of cancer cells to radiotherapy and chemotherapy. A completely sensitive response is ideal but rare, while radiotherapy and chemotherapy are usually impeded by acquired or intrinsic resistance. Acquired resistance can be loosely divided into cancer stem cell (CSC)-mediated and non-CSC-mediated resistance. CSC-mediated resistance is attributed to the potential ability of CSCs to proliferate and differentiate, which results in regressed tumor recurrence or seeds new tumors in a new place via metastasis. Non-CSC-mediated resistance factors include therapy stress-induced secondary mutations, tumor heterogeneity, altered endoplasmic reticulum (ER) stress, autophagy, drug distribution and metabolism, and so on. The intrinsic resistance mechanisms are similar to those mediating acquired resistance, but the difference is that the characteristics in intrinsic resistance are inherent and not induced by therapy stress

**Fig. 3:**
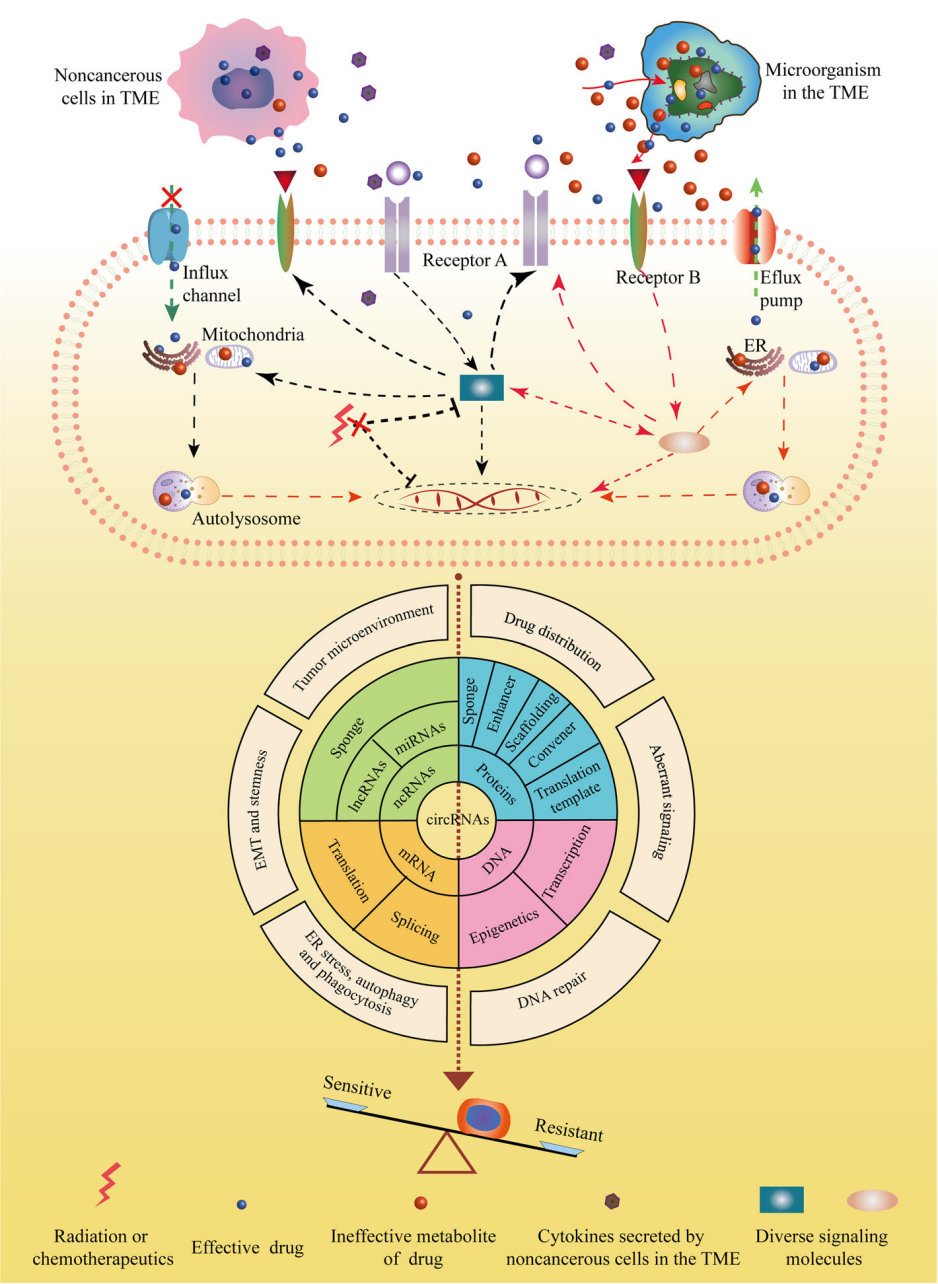
Roles of circRNAs in mechanisms mediating radioresistance and chemoresistance of cancers. In tumor microenvironment (TME), microorganisms modulate the critical chemical structures or local concentrations of drugs. In addition to regulating the distribution of drugs, noncancerous cells, such as CAFs and CAMs, can secrete soluble factors to promote therapy resistance. Remodeling of transporters or channels responsible for drug flux could result in decreased intracellular drug concentration. Amplification of targets or activation of aberrant downstream signals also impedes therapeutic efficacy by inducing ER stress, autophagy, mitophagy, stemness and DNA repair

The efficiency of cancer therapy is usually limited by intrinsic and acquired resistance (Fig. 2). CircRNAs widely influence cancer characteristics, such as enhanced DNA repair, reduced drug accumulation, target gene amplification and a favorable tumor microenvironment (TME), all of which are important for therapy resistance (Fig. 3).

### Increased drug efflux transporters or decreased influx channels

Reduced drug concentration is a primary cause of chemoresistance. This may result from drug sequestration in intracellular vesicles and compartments, increased drug efflux or decreased drug influx. These changes may be attributed to remodeling of drug channels and transporters, including ATP-binding cassette (ABC) family proteins, solute carriers and volume-regulated anion channels (VRACs) [12, 32–34].

ABC transporters can efficiently transport their corresponding substrates, such as chemotherapeutics, hormones and lipids, to the extracellular compartment, specific organelles and exosomes. Through these processes, the absorption, metabolism and activity of these substrates will be modified. Therefore, ABC transporters are promising targets for reversing multidrug resistance (MDR) [35–37]. Circ-ABCB10 (hsa_circ_0008717), derived from a region in the ABCB10 gene, is highly expressed in breast cancer, epithelial ovarian cancer (OC) and HCC, which correlates with patient survival. However, whether it affects the expression and function of ABC transporters is worth further investigation [38, 39]. Silencing of the circRNA PVT1 (circPVT1) decreases the expression of ABC subfamily B member 1 (ABCB1 or P-gp) and ABCC1 [40, 41]. CircSETD3 could bind to miR-520 to increase the level of ABCG2, leading to a decreased intracellular accumulation of gefitinib [36].

Platinum-based drugs enter the cell partially through passive diffusion across the cellular membrane and partially through special channels such as VRACs. Leucine-rich repeat-containing protein 8C (LRRC8) heteromers are essential components of VRACs. Chronic myeloid leukemia (CML) cells deficient in LRRC8A or LRRC8D were proved to be more resistant to carboplatin and cisplatin than cells with these proteins [33, 42].

### Enhanced epithelial-to-mesenchymal transition (EMT) and stemness

CiRS-7 (CDR1as or CDR1NAT), a cancer suppressor derived from the lncRNA LINC00632 transcripts, is upregulated in osteosarcoma (OS) and esophageal squamous cell carcinoma (ESCC). It is positively associated with cancer progression and promotes EMT by regulating miR-7 and miR-876-5p [43, 44]. EMT is an epigenetic program, through which the cells lose their epithelial phenotypes and gain mesenchymal characteristics. EMT-inducing transcription factors such as twist and snail, contain complementary binding sites to promoters of ABC transporters. They not only enhance EMT but also elevate the expression of ABC transporters. Silencing of these EMT inducers successfully reverses EMT-related MDR in breast cancer cells [12]. CircSMAD2 is capable of suppressing EMT by reducing miR-629 expression, which is a potential target for enhancing therapy sensitivity [45].

Circ_001680 and circ-NOTCH1 are increased in colorectal cancer (CRC) and gastric cancer (GC), respectively. Both promote the cancer stem cell (CSC) population in cancer [46, 47]. Failing to eradicate cancer cells that acquire stemness via activation of EMT or senescence is another reason for compromised therapy outcomes in cancer [48–51]. CSCs are capable of being dormant for a very long time to escape from harmful stress, such as radiotherapy and chemotherapy. Once the harmful stress is removed, CSCs may differentiate and proliferate at the primary lesion or metastasize and then invade other organs, leading to regressed tumor recurrence or metastasis [13, 16, 52].

### Copy number alterations of target genes and activation of bypass pathways or downstream signals

Heterogeneity is another major characteristic of cancer [11]. It promotes cancer resistance to therapy and even retards immunotherapy responses [53]. Heterogeneity indicates that various types of cells are present in an individual tumor, while a specific signal may play a predominant role in different cells and in different stages of cancer [11, 54–58]. Elevation of PD-L1 is a primary obstacle in cancer immunotherapy. CiRS-7 and circRNA-002178 were proven to increase the accumulation of PD-L1 at the cell membrane [59–61]. Extrachromosomal DNA (ecDNA) without centromeres will not be averagely transmit to new generations, which is a critical cause for heterogeneity. Amplification of the dihydrofolate reductase gene in ecDNA induces methotrexate resistance [62]. In contrast, the cells will become resistant to EGFR inhibitors if the mutant epidermal growth factor receptor (EGFR) is depleted from ecDNA. This causes the reemergence of tumors following drug withdrawal [63].

PI3K inhibitors are reported to not only induce the activation of the oncogene Myc but also stimulate the amplification of PIK3CA, both of which reduce cancer cell sensitivity [64]. Osimertinib is able to increase the phosphorylation of AXL in EGFR-mutated non-small cell lung cancer (NSCLC) cells, which subsequently activate HER3, MET, EGFR and the corresponding downstream signals. All of these oncological signals reduce the responses of cancers to osimertinib [65]. Similarly, inhibition of PI3Kα also increases AXL expression, which induces chemoresistance through transactivation of the EGFR/PKC/mTOR axis independent of PI3K [66].

STAT3 and AKT are strongly associated with carcinogenesis by enhancing the proliferation and reducing the apoptosis of tumor cells [67–69]. The Skp2/AKT axis is capable of inducing the phosphorylation of programmed cell death protein 4 (PDCD4), a protein that promotes the DNA damage response and apoptosis. Blockade of this action favors cancer cells resistant to radiotherapy [15]. Under chemotherapy stress, the feedback activation of STAT3 by the IL-6/JAK1 or FGFR/PI3K pathway prevents cancer cells from undergoing the apoptosis induced by inhibitors of receptor tyrosine kinases (RTKs), such as MEK or KRAS [68]. Hsa_circ_0017250, derived from the AKT gene, encodes a protein that can inactivate AKT and enhance cell sensitivity to radiation [25].

### Remodeling of ER stress, autophagy and phagocytosis

Both activating and inhibiting ER stress can overcome chemoresistance and radioresistance in cancer [70–72]. This indicates that the roles of ER stress in cell fate are multifaceted and context-dependent. ER stress is also a potent inducer of autophagy that manipulates the sensitivity of cancers [73]. For instance, imatinib induces autophagy that facilitates chemoresistance [74]. However, autophagy is also a double-edged sword. Mild autophagy favors cell survival under normal or mild stress conditions, while excessive autophagy leads to cell death. Therapy resistance may be attributed to autophagy-mediated changes in oxidative stress and autophagy-supplied key metabolites for the maintenance of stemness during dormancy [75–78]. Interestingly, circEIF6 promotes cisplatin-induced autophagy and enhances chemoresistance in GC cells [79].

Following doxorubicin (DOX) treatment, the induced senescent cancer cells can expand, approach and then completely engulf the neighboring cells. The engulfed cells are transported to the lysosomes for degradation, which is similar to autophagy in terms of supplying energy and key metabolites. This reduces chemotherapy responses by mechanisms such as preventing tumor cell death and leads to tumor relapse [14].

### Enhanced or weakened DNA repair ability

Inducing DNA damage is another primary action of radiotherapy and chemotherapy that usually causes cell death. However, DNA repair is able to reduce the sensitivity of cancer cells to therapy. Mutations in the BRCA1 and BRCA2 genes, two critical enzymes for homologous recombination (HR) or mismatch repair (MMR) DNA repair, can promote the progression of breast cancer and OC [80, 81]. Due to their limited DNA repair ability, cells with certain BRCA1 or BRCA2 mutations are supposed to be sensitive to cell death stimuli such as cisplatin, poly ADP-ribose polymerase (PARP) inhibitors and radiation [82, 83]. However, hsa_circ_0000199 has the potential to activate the DNA repair molecule histone family member X (H2AX), leading to resistance to cisplatin [81, 84].

Furthermore, the expression of dynein light chain 1 protein (DYNLL1) is positively correlated with progression-free survival (PFS) in platinum-based chemotherapy in patients with BRCA1-mutated ovarian carcinoma. Once DYNLL1 is inactivated, the nuclease activity of MRE11, a double-strand break repair protein, will be restored to repair errors in DNA, even without BRCA1 coordination. This further confers resistance to platinum drugs and PARP inhibitors in BRCA1-mutant cells [83]. Interestingly, therapeutic stresses such as EGFR/BRAF inhibitors decrease the levels of MMR and HR DNA repair genes, meanwhile increase the error-prone polymerases. These actions prompt adaptive DNA mutability and ultimately restrain chemotherapy efficacy by conferring cancer cell resistance [85].

### Favorable tumor microenvironment

The TME is a complex ecosystem containing the microbiota, an acidic pH, inflammatory factors, matrix metalloproteinases, extracellular matrix (ECM), cancer-associated fibroblasts (CAFs), tumor-associated macrophages (TAMs) and so on. The TME is even different between the primary tumor and the corresponding metastases, which also affects cancer cell sensitivity [11, 86]. Inflammatory factors such as IL-7 can reduce the expression of ABCG2 and resensitize NSCLC cells to cisplatin [87]. The hypoxic TME induces excessive mitochondrial fission in natural killer (NK) cells. This reduces NK cell viability and immunosurveillance [88]. Tumor cell-secreted PRSS (a serine protease) not only promotes angiogenesis and cancer cell invasion but also induces resistance to monoclonal antibodies such as cetuximab and bevacizumab by cleaving them into inactive forms [89]. The microorganisms in the TME decrease the efficacy of chemotherapeutics in various manners. For example, Gammaproteobacteria, a common bacterial species in pancreatic cancer tissues, lead to poor responses by metabolizing gemcitabine into inactive metabolites in cancer cells [90].

Before cancer cells, TAMs will infiltrate into irradiated normal tissues under radiotherapeutic stress. Then, TAMs secrete soluble factors such as inflammatory protein 1 beta to recruit cancer cells, promoting cancer metastasis and recurrence following radiotherapy [91]. Soluble factors from TAMs also have the potential to induce the expression of cytidine deaminase (a metabolism enzyme) in cancer cells, which reduces the efficacy of gemcitabine [90].

Similar to TAMs, CAFs render tumor cells resistant to 5-FU, which is correlated with the poor cumulative survival of GC patients. The responses of lung cancers with podoplanin-positive CAFs to EGFR tyrosine kinase inhibitors (TKIs) are worse than those of lung cancers with podoplanin-negative CAFs [92, 93]. There are many other factors in the TME that can affect drug sensitivity, such as an aberrant vasculature failing to transport drugs. With diverse functions, circRNAs such as ciRS-7 have already been shown to regulate various characteristics of the TME, which has been proven to regulate the sensitivity of several types of cancer to chemotherapy [26, 94–98].

## Advances in the understanding of circRNA-mediated radioresistance and chemoresistance in cancer

Although the underlying mechanisms of therapy resistance have been extensively studied, it is still one of the biggest obstacles in cancer treatment. Recently, circRNAs have attracted intensive interest from oncologists due to their potential functions in tumor biology. There have been dozens of reports on the actions of circRNAs in therapy resistance (Tables 1 and 2), of which only several studies are about the role of circRNAs in radiotherapy responses (Table 1).

**Table 1:** Alterations of circRNAs in radiotherapy resistance

Cancer	Numbers of alterations/name of circRNA	Alteration	Target	References
Glioma	hsa_circ_0017250	↓	AKT protein	[25]
	48	↓	–	[99]
	63	↑		
Nasopharyngeal carcinoma	1558	↓	–	[100]
	1042	↑		
	circRNA_000543	↑	miR-9	[101]
Oral squamous cell carcinoma	circATRNL1	↓	miR-23a-3p	[102]
Esophageal cancer	17	↓	Enriched in Wnt signaling pathway	[103]
	57	↑		
	circVRK1	↓	miR-624-3p	[104]
	circRNA_100367	↑	miR-217	[105]
Hepatocellular carcinoma	cZNF292	Increased under hypoxia	SOX9 protein	[106]
Colorectal cancer	47	↓	–	[107]
	24	↑		
	circCCDC66	↑	miR-338-3p	[108]
Cervical cancer	76	↑	–	[109]
	77	↓		

Notes: ↑ means increased; ↓means decreased; −- means not reported yet

**Table 2:** Altered circRNAs in chemoresistant cancer cells

System	Cancer	Number of alterations/name of circRNA	Alteration	Target	Related drugs	References
Musculoskeletal system	Osteosarcoma	hsa_circ_0001258	↓	miR-744-3p	Doxorubicin	[110]
		circ-LARP4		miR-424	Cisplatin, doxorubicin	[111]
		has_circ_0004674	↑	–	Doxorubicin	[112]
		circPVT1			Methotrexate, doxorubicin, cisplatin, ifosfamide	[40]
Respiratory System	Metastatic Nasopharyngeal carcinoma	circCRIM1	↑	miR-422a	Paclitaxel	[113]
	Lung cancer	7966	↑	–	Osimertinib	[114]
		7538	↓			
		2909	↑	–	Paclitaxel	[20]
		8372	↓			
		circESRP1	↓	miR-93-5p	Doxorubicin, cisplatin, etoposide	[115]
		circ_0002483	↓	miR-182-5p	Paclitaxel	[116]
		hsa_circ_0004015	↑	miR-1183	Gefitinib	[117]
		circSETD3		miR-520 h		[36]
		CCDC66		–	Cisplatin	[118]
		circ_0076305		miR-296-5p	Cisplatin	[119]
		circFGFR1		miR-3 81-3p	PD-1 antibody	[120]
		circPVT1		miR-145-5p	Cisplatin, pemetrexed	[41]
		ciRS-7		–	Pemetrexed, cisplatin	[121]
Hematopoietic system	Leukemia	circ_100053	↑	–	Imatinib	[122]
		circMYBL2		PTBP1 protein	Quizartinib	[24]
		crcPAN3		miR-153-5p and miR-183-5p	Doxorubicin	[123, 124]
		circBA9.3		–	Imatinib, nilotinib, dasatinib	[125]
		circ_0009910		miR-34a-5p	Imatinib	[126]
Endocrine system	Thyroid carcinoma	circEIF6	↑	miR-144-3p	Cisplatin	[79]
	Pancreatic cancer	chr14: 101402109–101464448+, chr4: 52729603–52780244+	↑	miR-145	Gemcitabine	[127]
		26	↑	–		[19]
		55	↓			
	Cervical cancer	circMTO1	↑	miR-6893	Cisplatin	[128]
		hsa_circ_0023404		miR-5047		[129]
	Breast cancer	circ 0006528	↑	miR-7	Doxorubicin	[130]
		ciRS-7		miR-7	Cisplatin, 5-FU, tamoxifen	[131–133]
		circRNA-MTO1	↓	TRAF4 protein	Monastrol	[134]
		hsa_circ_0025202		miR-182-5p	Tamoxifen	[135]
		circBMPR2		miR-553		[136]
		circKDM4C		miR-548p	Doxorubicin	[137]
Digestive system	Oral carcinoma	hsa_circ_0005379	↓	–	Cetuximab	[138]
	Hepatocellular carcinoma	circRNA_101505	↓	miR-103	Cisplatin	[31]
	Gastric cancer	hsa_circ_0000199	↑	miR-198	Cisplatin	[84]
		hsa_circ_0081143		miR-646		[139]
		circFN1		miR-182-5p		[140, 141]
		circPVT1		miR-124-3p	Paclitaxel	[141]
	Colorectal cancer	773	↑	–	5-FU and oxaliplatin	[142]
		732	↓			
		34	↓			[29]
		105	↑			
		circ_001680	↑	miR-340	Irinotecan	[46]
		ciRS-122		miR-122	Oxaliplatin	[143]
Urinary system	Renal cancer	hsa_circ_0035483	↑	miR-335	Gemcitabine	[144]
	Bladder cancer	hsa_circ_0000285	↓	–	–	[145]
		ciRS-7		miR-1270	Cisplatin	[95]
		circELP3	↑	–	–	[96]
	Prostate cancer	circRNA17	↓	miR-181c-5p	Enzalutamide	[146]
		588	↓	–		[147]
		278	↑			
		hsa_circ_0022392 and hsa_circ_0000326	↓	–	–	[148]
		hsa_circ_0001275 and hsa_circ_0001721	↑	–	–	
Reproductive system	Ovarian cancer	ciRS-7	↓	miR-1270	Cisplatin	[26]
		circCELSR1	↑	miR-1252	Paclitaxel	[149]
Miscellaneous	Myeloma	hsa_circ_0007841	↑	–	–	[150]
	Melanoma	ciRS-7	↓	–	–	[151]

Notes: ↑ means increased; ↓means decreased; −- means not reported yet

### CircRNAs in cancer radioresistance

#### Glioma

CircATP8B4 is one of the increased circRNAs (Table 1) in radioresistant glioma, and it sponges miR-766, which is probably the mechanism through which circATP8B4 reduces radiation sensitivity [99]. The blood-brain barrier (BBB) prevents many drugs from entering the brain, leading to a poor response of glioma to chemotherapy. An in vitro study indicated that the circRNA DENND4C (cDENND4C), as an miR-577 sponge, increases mimicked BBB permeability by reducing the expression of tight junction-related proteins. This allows more DOX across mimicked BBB in vitro, leading to apoptosis of glioma cells [152]. However, whether this phenomenon also exists in vivo has still not been studied. Thus, radiotherapy is still the preferred strategy for brain cancers to date. Three circRNAs generated from the AKT3 gene (hsa_circ_0017250, hsa_circ_0112784 and hsa_circ_0112781) are decreased in glioma tissues. Hsa_circ_0017250, containing a complete ORF, encodes a protein named AKT3-174aa. AKT3-174aa acts as a dominant-negative variant of AKT and inhibits the phosphorylation of AKT^T308^, thus enhancing the sensitivity of glioma cells to radiation [25].

#### Nasopharyngeal carcinoma

In radioresistant nasopharyngeal carcinoma (NPC) cells, 2600 altered circRNAs have been identified, 1042 of which were upregulated and 1558 of which were downregulated. Hsa_circRNA_006660 was further proven to enhance radiosensitivity via miR-1276 [100]. CircRNA_000543 is elevated in NPC tissues and is much higher in radioresistant samples than in radiosensitive samples. Silencing it sensitizes NPC cells to irradiation. It was further demonstrated that circRNA_000543 sponges miR-9 and then increases transcription of platelet-derived growth factor receptor beta (PDGFRB), leading to poorer overall survival of NPC patients [101].

#### Oral squamous cell carcinoma

CircATRNL1, derived from the ATRNL1 gene, is one of the dysregulated circRNAs that is reduced in oral squamous cell carcinoma (OSCC) cells, and its level is lower following irradiation. When circATRNL1 is upregulated, the radiosensitivity of OSCC cells is enhanced. This is because it sponges miR-23a-3p to increase the transcription of PTEN and then decreases the phosphorylation of AKT, suggesting that autophagy may be involved [102].

#### Esophageal cancer

Su et al. identified 57 circRNAs that were upregulated and 17 that were downregulated in radioresistant ESCC cells. Wnt signals were revealed to be the most enriched downstream pathways of these altered circRNAs [103, 153]. The level of circRNA_100367 is increased in radioresistant ESCC cells. It binds to miR-217 to promote Wnt3-mediated EMT, resulting in reduced sensitivity of cancer cells to radiation [105]. CircVRK1 is decreased in ESCC tissues, and patients with low level of circVRK1 have a poor prognosis. This is probably because circVRK1, as a sponge of miR-624-3p, decreases the PTEN/PI3K-mediated activity of AKT and inhibits EMT, which enhances the sensitivity of ESCC cells to radiotherapy [104].

#### Hepatocellular carcinoma

cZNF292 is upregulated in HCC cells under hypoxia independently of HIF-1α. Silencing cZNF292 inhibits radiation-induced γ-H2AX foci formation due to its binding with SOX9 protein to inhibit the activity of the β-catenin-mediated DNA repair pathway. This ultimately enhances the sensitivity of HCC cells to radiotherapy [106].

#### Colorectal cancer

A microarray analysis identified 47 upregulated circRNAs and 24 downregulated circRNAs in 5-FU- and radiation-resistant CRC cells. Bioinformatics pathway analysis revealed that these circRNAs were related to miRNAs and cancer-associated signals, such as the Wnt pathway [107]. CircCCDC66 is increased in CRC tissues and is much higher in radioresistant tissues than in radiosensitive ones and is associated with decreased levels of miR-338-3p. Since circCCDC66 sponges miR-338-3p, silencing circCCDC66 inhibits cell viability and enhances CRC cell radiosensitization via caspase-3 [108].

#### Miscellaneous

It was identified that 61 circRNAs are upregulated and 97 are downregulated in human embryonic kidney (HEK) 293 T cells following radiation. The circRNA-miRNA-mRNA network analysis revealed that the altered circRNAs were suggested to be involved in EGFR and mTOR signaling, which is worth further studies in cancer cells [154]. Duo et al. identified 76 upregulated and 77 downregulated circRNAs in irradiated HeLa cells. However, whether these altered circRNAs are responsible for resistance enhancers such as MAPK signals has still not been studied [109, 155].

### CircRNAs in cancer chemoresistance

#### Musculoskeletal system cancer: osteosarcoma

Eighty circRNAs were identified as differentially expressed between chemoresistant and chemosensitive OS cells via circRNA profiling assays (20,836 circRNAs). Bioinformatics analysis showed that the altered circRNAs in chemoresistant OS cells were involved in several vital signaling pathways responsible for drug resistance, such as pathways featuring ABC transporters and VEGF. Hsa_circ_0001258 is decreased while hsa_circ_0004674 is increased in chemoresistant OS cells and tissues [110, 112]. Hsa_circ_0001258 sponges miR-744-3p to increase the expression of GSTM2. Inhibition of this axis induces resistance of OS cells to DOX [110]. Circ-LARP4 is downregulated in OS and negatively correlated with the Enneking stage and survival due to its ability to increase the sensitivity of tumor cells to cisplatin and DOX by sponging miR-424 [111].

Reduction of circ_001569 enhances the sensitivity of OS to cisplatin, DOX and methotrexate. Although circ_001569 overexpression increases the expression of p-GSK-3β and β-catenin, the Wnt/β-catenin agonist LiCl can restore the sensitivity of cancer cells to only cisplatin but not to DOX and methotrexate, suggesting that other mechanisms may be involved [138]. CircPVT1 is increased in OS tissues, serum, lung metastases and chemoresistant cells, which is correlated with poor prognosis. Knockdown of CircPVT1 partially reverses the resistance of OS cells to DOX and cisplatin by decreasing the expression of MDR protein 1 (MDR1 or ABCB1). It is speculated that circPVT1 may act as a sponge of miRNAs such as miR-125 to contribute to chemoresistance, which has not yet been validated [18, 40].

#### Respiratory system cancer

##### Nasopharyngeal carcinoma

CircCRIM1 was upregulated in highly metastatic NPC cells and tissues with distant metastasis. NPC patients with higher level of circCRIM1 has poorer therapeutic responses to docetaxel-based therapy, overall survival, disease-free survival and distant metastasis-free survival rates. CircCRIM1 is able to sponge miR-422a to increase mRNA and protein levels of FOXQ1. Through this, circCRIM1 promotes NPC cell metastasis and EMT. Silencing of it significantly enhances inhibitory efficacy of paclitaxel on metastasis in NPC cells [113].

##### Lung cancer

In osimertinib-, DOX- or paclitaxel-resistant lung cancer cells, thousands of altered circRNAs have been identified (Table 2) [20, 114, 115]. Similar to its role in radioresistant cervical cancer cells, hsa_circ_0004015 is also increased and confers resistance to gefitinib by regulating miR-1183/PDPK1 in NSCLC cells [109, 116, 117]. Circ_0076305 and circFGFR1 (hsa_circ_0084003) are elevated while circ_0002483 is decreased in NSCLC cells, which trends are more significant in the cisplatin- or paclitaxel-resistant NSCLC cells, respectively [116, 119]. Binding of circ_0076305 to miR-296-5p upregulates STAT3 and promotes cisplatin resistance [119]. Circ_0002483 reduces cell sensitivity to paclitaxel via the miR-182-5p/GRB2/FOXO1 axis [116]. Upregulation of hsa_circ_0071799 affects miR-141, while downregulation of hsa_circ_0091931 affects miR-34c-5p, both of which may mediate osimertinib resistance [20].

CircCCDC66 and hsa-circRNA-002178 are highly expressed in lung adenocarcinoma (LUAD) cells [60, 118]. CircCCDC66 is positively regulated by FAK (a marker of EMT) and HGF/c-Met, while it is negatively regulated by the acetylcholine receptor nAchR7α. However, reduction of circCCDC66 does not affect the resistance of H1975 cells to gefitinib or erlotinib but inhibits EMT and reduces cell sensitivity to cisplatin [118]. Hsa-circRNA-002178 enhances PD-L1 expression by sponging miR-34 to induce T cell exhaustion. Furthermore, it could also be transferred into CD8+ T cells to induce PD-1 expression via exosomes [60]. The elevation of circFGFR1 reduces PD-1-positive lung cancer cell and CD8+ T cell frequency by regulating the miR-381-3p/C-X-C motif chemokine receptor 4 (CXCR4) axis, which is responsible for reduced sensitivity to anti-PD-1 antibodies [120].

CircSETD3 is increased in the plasma of gefitinib-resistant NSCLC patients and cells. It could directly bind to miR-520 to increase the expression of ABCG2, leading to a decreased intracellular accumulation of gefitinib [36]. The expression levels of ciRS-7 and circPVT1 are increased in pemetrexed- and cisplatin-resistant lung cancer cells. They reduce the efficiency of neoadjuvant chemotherapy (pemetrexed and cisplatin) by inhibiting the EGFR/PI3K pathway and the miR-145-5p/multidrug resistance protein 1 (MRP1 or ABCC1) axis, respectively [41, 121].

CircRNA_101505 confers HCC cells with resistance to cisplatin by regulating miR-103 [31]. MiR-103 is downregulated in lung cancer cells, and upregulation of miR-103 enhances the sensitivity of NSCLC cells to gefitinib [156]. However, whether circRNA_101505 is involved in MET- and miR-103-conferred gefitinib resistance in lung cancer still needs further evaluation. Circular RNA epithelial splicing regulatory protein-1 (cESRP1) is decreased in clinical small cell lung cancer (SCLC) tissues and DOX- and cisplatin/etoposide-resistant lung cancer cells. cESRP1 sponges miR-93-5p and activates Smad7/p21-TGFβ-mediated EMT. Silencing cESRP1 caused chemoresistance and poor prognosis in SCLC patients [115].

#### Hematopoietic system cancer: leukemia

The introduced fusion of circRNA M9 reduces the sensitivity of K562 cells to arsenic trioxide (ATO) [157]. Circ_100053 and circ_0009910 are increased in imatinib-resistant leukemia cells [122, 126]. Circ_0009910 sponges miR-34a-5p to elevate ULK1 and trigger autophagy, thus contributing to the chemoresistance of CML [126]. As a transcriptional accessory of BCR-ABL1, circBA9.3 is positively correlated with BCR-ABL1 in imatinib-resistant patients. Introducing exogenous circBA9.3 into TKI-sensitive K562 cells induces the resistance of CML not only to imatinib (1st generation TKI) but also to nilotinib and dasatinib (2nd generation TKIs). This probably occurs through increased expression of ABL1 and BCR-ABL1 at the posttranscriptional level [125].

CircPAN3 is increased in DOX-resistant acute myeloid leukemia (AML) cells and bone marrow cells from relapsed patients, while several target miRNAs are decreased. CircPAN3 has the potential to activate AMPK and inhibit mTOR, which subsequently induces autophagy. Meanwhile, circPAN3 binding to miR-153-5p and miR-183-5 increases the activity of X-linked inhibitor of apoptosis protein (XIAP). Without affecting basal apoptosis, knockdown of circPAN3 restores the sensitivity of AML cells to chemotherapeutics [123, 124]. CircMYBL2 could directly interact with the PTBP1 protein, which subsequently promotes the expression of FLT3 kinase at the translational level and phosphorylation of FLT3 kinase. These effects are why silencing circMYBL2 reduces proliferation, promotes differentiation and enhances cell sensitivity to quizartinib in FLT3-ITD AML cells [24].

#### Endocrine system cancer

##### Thyroid carcinoma

CircEIF6 is highly expressed in papillary thyroid carcinoma (PTC) tissues and negatively regulates miR-144-3p expression. Exposure of TPC1 and BHT101 cells to cisplatin further increases circEIF6 and decreases miR-144-3p expression. CircEIF6 upregulation promotes cisplatin-induced autophagy; thus, it enhances the resistance of PTC cells to cisplatin via the miR-144-3p/TGF-α axis [79].

##### Pancreatic cancer

Xu et al. identified that 26 circRNAs were upregulated and 55 were downregulated in gemcitabine-resistant pancreatic cancer cells. Further analysis showed that several markedly changed circRNAs were potentially involved in oncogenic pathways such as the MAPK pathway [19]. Another study showed that two circRNAs (Table 2) were significantly increased while miR-145 was decreased in plasma from gemcitabine-nonresponsive patients and in gemcitabine-resistant PANC-1 (PANC-1/GEM) cells. Knockdown of them circRNAs effectively restores the sensitivity of PANC-1/GEM cells to gemcitabine, probably via miR-145 [127].

##### Cervical cancer

CircRNA-MTO1 (hsa-circRNA-007874) and hsa_circ_0023404 are upregulated in cervical tumor cells, and both promote tumorigenesis and chemoresistance to cisplatin [128, 129, 158]. Sponging of miR-6893 by circMTO1 induces S100A1 expression and autophagy, leading to reduced chemosensitivity [128].

##### Breast cancer

Circular RNA angiomotin-like 1 (circAMOTL1) could decrease paclitaxel-induced apoptosis by enhancing AKT signaling [159]. Hsa_circ_0001839 (circKDM4C) is decreased while hsa_circRNA_0003218 (circBMPR2) is increased in breast cancer tissues, especially in metastatic or chemoresistant tissues [136, 137]. Silencing circKDM4C promotes EMT while decreases apoptosis and the sensitivity of breast cancer cells to DOX by sponging miR-548p [137]. Circ_0006528 is increased in DOX-resistant breast cancer cells, while silencing of it enhances cell chemosensitivity by increasing miR-7-5p and Raf1 expression [130].

The expression level of ciRS-7 and REGγ are higher while that of miR-7 is lower in breast cancer than in normal tissues, which trends are significant following chemotherapy. CiRS-7 is negatively correlated with the outcome of chemotherapy in breast cancer, suggesting that ciRS-7 may contribute to therapy resistance [131]. Silencing ciRS-7 increases miR-7 and decreases REGγ expression. Indeed, the ciRS-7/miR-7 axis could mediate both cisplatin and 5-FU resistance in breast cancer cells [3, 131, 132]. CircRNA-MTO1 is decreased in monastrol-resistant breast cancer cells. Overexpression of circRNA-MTO1 inhibits viability and restores cell sensitivity to monastrol by reducing Eg5 translation and sequestering TRAF4 from the Eg5 gene [134]. Hsa_circ_0025202 enhances the sensitivity of breast cancer cells to tamoxifen as a sponge of miR-182-5p to upregulate FOXO3a at both the mRNA and protein levels [135]. CircBMPR2 is reduced in metastatic breast cancer cells. Moreover, its downregulation results in the resistance of tumor cells to tamoxifen due to the enhanced EMT mediated by the miR-553/ubiquitin-specific protease 4 (USP4) axis [136].

#### Digestive system cancer

##### Oral carcinoma

Hsa_circ_0005379 is downregulated in oral carcinoma tissues, while its elevation reduces cell proliferation, induces apoptosis and enhances the sensitivity of cancer cells to cetuximab. This is probably attributed to the function of hsa_circ_0005379 as an upstream inhibitor of EGFR- and EMT-related signals [138].

##### Hepatocellular carcinoma

Circ_0003418 and circRNA_101505 are decreased in HCC tissues, while circ-SMARCA5 is reduced in intrahepatic cholangiocarcinoma (ICC) [31, 160, 161]. Silencing circ_0003418 promotes cisplatin resistance, probably though miR-7- and miR-383-mediated effects on the Wnt/β-catenin pathway, but this mechanism still needs further investigation [161]. CircRNA_101505 is especially lower in cisplatin-resistant HCC cells than in sensitive cells [31]. HCC or ICC patients with a lower level of circRNA_101505 or circ-SMARCA5 have a shorter overall survival period than patients with higher levels of these circRNAs [31, 160]. Overexpression of circRNA_101505 increases its ability to sponge miR-103 and subsequently elevates the expression of the tumor suppressor oxidored-nitro domain-containing protein 1 (NOR1), which sensitizes HCC cells to cisplatin-induced apoptosis [31]. The upregulation of circ-SMARCA5 could enhance the sensitivity of ICC cells to cisplatin and gemcitabine [160].

##### Gastric cancer

Similar to its levels in OS, circPVT1 is also increased in paclitaxel-resistant GC tissues and cells. It confers paclitaxel resistance in GC cells by sponging miR-124-3p to upregulate zinc-finger E-box binding homeobox 1 (ZEB1) levels [141]. Downregulation of ciRS-7 in GC cells increases miR-7-5p levels and subsequently reduces REGγ expression, by which the toxicity of diosbulbin-B is enhanced [162].

Hsa_circ_0000199, circFN1 (hsa_circ_0058147) and hsa_circ_0081143 are predictive biomarkers of the sensitivity of GC patients to cisplatin, and they are upregulated in cisplatin-resistant GC cells [84, 139]. CircFN1 reduces GC cell apoptosis by sponging miR-182-5p in vitro and in vivo [140]. In addition to its status in cisplatin-resistant OC cells, PIK3R1, a regulatory subunit of PI3K, is also increased in cisplatin-resistant cells. Hsa_circ_0000199 sponges miR-198 to upregulate PIK3R1 at both the mRNA and protein levels. This further activates PI3K/AKT/γH2AX pathway-mediated DNA repair and confers cells with cisplatin resistance [84, 163]. Silencing of hsa_circ_0081143 makes cells more sensitive to cisplatin, probably by releasing miR-646, which reduces CDK6 in GC tissues [139].

##### Colorectal cancer

Hundreds of altered circRNAs have been identified in CRC cells resistant to 5-FU and oxaliplatin or the exosomes derived from these resistant cells (Table 2). The circRNAs contained in exosomes could be transferred into cocultured cells in vitro, indicating their potential to be transported into adjacent or distant cells to confer chemoresistance in vivo, but this mechanism still requires further evaluation [29, 142]. Bioinformatics analysis further showed that the altered circRNA-mediated signals were enriched in several cancer-related pathways. For instance, hsa_circRNA_103306/miR-370-3p has the potential to regulate drug metabolism, while has_circ_32883/miR-130b probably affects drug resistance via the PI3K/AKT pathway [142]. Hsa_circ_32883 and ciRS-122 (hsa_circ_0005963) are upregulated in chemoresistant CRC cells [142, 143]. CiRS-122 can be delivered into chemosensitive cells via oxaliplatin-resistant cells-secreted exosomes. Then, it promotes glycolysis and cell resistance to oxaliplatin by sponging miR-122 to upregulate the M2 isoform of pyruvate kinase (PKM2) level [143]. Circ_001680 is increased in CRC tissues. In addition to enhancing proliferation and migration, it also triggers irinotecan resistance via sponging miR-340 to reduce BMI1 transcription, which supports the population of CSCs [46].

#### Urinary system cancer

##### Renal cancer

Hsa_circ_0035483 is one of the most highly expressed circRNAs in renal clear cell carcinoma (RCC) tissues and cells, while its downstream target miR-335 displays the opposite trend. This inverse pattern is more obvious in the cells treated with gemcitabine. It was further demonstrated that the expression of hsa_circ_0035483 facilitates gemcitabine-induced autophagy and enhances the resistance of RCC to gemcitabine by regulating the hsa-miR-335/cyclin B1 axis [144].

##### Bladder cancer

CircELP3 is increased and hsa_circ_0000285 is decreased in bladder cancer tissues or serum. Downregulating circELP3 reduces the sphere-forming ability of bladder cancer stem-like cells under hypoxic conditions. Moreover, hsa_circ_0000285 is exceptionally lower in cisplatin-resistant patients than in cisplatin-sensitive patients [96, 145]. Contrary to its high expression in breast cancer, ciRS-7 is expressed at low levels in bladder cancer [131, 164]. After induced overexpression of exogenous ciRS-7 in bladder cancer cells, proliferation, invasion and migration are markedly reduced, while cisplatin sensitivity is enhanced. This results from ciRS-7 acting as a sponge of miR-1270 to increase the mRNA level of apoptotic protease activating factor-1 (APAF1) [95].

##### Prostate cancer

Recently, Greene et al. identified 278 circRNAs that were increased and 558 that were decreased in enzalutamide (ENZ)-resistant prostate cancer cells. Hsa_circ_0004870, derived from the RBM39 gene, is one of the significantly down-regulated circRNAs. It likely enhances chemoresistance by regulating the splicing of U2 small nuclear RNA auxiliary factor 65 (U2AF65) [147]. Silencing of circRNA Foxo3 (circFoxo3 or hsa_circis_0006404) increases the survival of androgen-enhanced prostate cancer cells and represses apoptosis while enhancing resistance to docetaxel. This is probably because the reduced activity of the circFoxo3/Foxo3 axis may lead to enhanced EMT [165]. CircRNA17 (hsa_circ_0001427), derived from the PDZ and LIM domain protein 5 (PDLIM5) gene, can increase and interact with miR-181c-5p to inhibit the transcription of androgen receptor variant 7 (ARv7). Due to ENZ-suppressed transcription of PDLIM5, circRNA17 is reduced in castration- and ENZ-resistant prostate cancer (CRPC) cells, which further grants cells resistance to ENZ [146].

#### Reproductive system cancer: ovarian cancer

Silencing of circ-ABCB10 promotes apoptosis of breast cancer cells and epithelial OC cells via decreased sponging of miR-1271, miR-1252 and miR-203 [38, 39]. Similar to its status in breast cancer, ciRS-7 is also decreased in tissues and serum exosomes derived from cisplatin-resistant OC patients [26, 95]. Exogenous expression of ciRS-7 distinctly inhibits the proliferation and migration of cisplatin-resistant OC cells while promotes cisplatin-induced apoptosis. CiRS-7 functions as a molecular sponge of miR-1270 to upregulate the transcription of suppressors of invasion (SCAI) [26]. Interestingly, circCELSR1 (hsa_circ_0063809), localized in the cytoplasm, is increased in paclitaxel-resistant OC tissues and cells, while miR-1252 is decreased in paclitaxel-resistant OC tissues and cells. It was further proven that circCELSR1 is negatively correlated with paclitaxel chemosensitivity by positively regulating FOXR2 expression by sponging miR-1252 [149].

#### Miscellaneous: myeloma and melanoma

Hsa_circ_0007841 is upregulated in bortezomib-resistant multiple myeloma (MM) cells and significantly correlated with the poor prognosis of MM patients [150]. CiRS-7 is downregulated in melanoma and negatively correlates with melanoma progression by interacting with the RBP IGF2BP3. Melanoma cells with lower levels of ciRS-7 are more sensitive to multiple MAPK pathway inhibitors and GPX4 inhibitors. However, silencing of ciRS-7 in melanoma cells with high ciRS-7 expression had no effect on cell sensitivity to the BRAF inhibitor dabrafenib and the GPX4 inhibitor RSL3 (1S,3R-RSL-3), suggesting that ciRS-7 levels are only a biomarker of chemotherapy response but not a critical mediator in melanoma [151].

## Conclusions and prospects

Radiotherapy and chemotherapy are two widely used strategies in cancer treatment. However, intrinsic and acquired resistance are still critical obstacles for therapy outcome. CircRNAs are tissue- and cell type-specific, as well as cancer- and drug/radiation response-specific, leading to different expression patterns of circRNAs and associated regulatory pathways upon therapy stress. With high distinct abundance and stability, circRNAs are promising targets for overcoming cancer resistance to radiation and chemotherapy.

Currently, research on the role of circRNAs in the resistance of cancers to radiotherapy and chemotherapy is still at the nascent stage. First, the available reports about circRNAs in cancer chemoresistance are limited to only a few classic drugs and a few kinds of cancers. Second, most of the reported circRNAs, even when restricted to circRNAs identified in cancer, still have not been studied in cancer therapy responses, especially in radioresistance, and countless unidentified circRNAs have yet to be studied. These limitations will be overcome gradually, and potential interventions targeting circRNAs are promising for overcoming the resistance of cancer to radiation and chemotherapy.

## Data Availability

Not applicable.

## Abbreviations

ABC: ATP-binding cassette
AML: Acute myeloid leukemia
APAF1: Apoptotic protease activating factor-1
AR: Androgen receptor
BBB: Blood-brain barrier
cESRP1: Circular RNA epithelial splicing regulatory protein 1
circRNAs: Circular RNAs
CRC: Colorectal cancer
CRPC: Castration-resistant prostate cancer
DOX: Doxorubicin
DYNLL1: Dynein light chain 1 protein
ecDNA: Extrachromosomal DNA
EGFR: Epidermal growth factor receptor
ENZ: Enzalutamide
ER: Endoplasmic reticulum
ESCC: Esophageal squamous cell carcinoma
EMT: Epithelial-to-mesenchymal transition
ecircRNAs: Exonic circRNAs
GC: Gastric cancer
H2AX: H2A histone family member X
HCC: Hepatocellular carcinoma
HR: Homologous recombination
ICC: Intrahepatic cholangiocarcinoma
LUAD: Lung adenocarcinoma
LncRNAs: Long ncRNAs
LRRC8: Leucine-rich repeat-containing protein 8C
MDR: Multidrug resistance
MDR1: MDR protein 1
miRNAs: MicroRNAs
MMR: Mismatch repair
MRP1: Multidrug resistance protein 1
ncRNAs: Non-coding RNAs
NK: Natural killer
NOR1: Oxidored-nitro domain-containing protein 1
NPC: Nasopharyngeal carcinoma
NSCLC: Non-small cell lung cancer
OC: Ovarian cancer
OS: Osteosarcoma
OSCC: Oral squamous cell carcinoma
PARP: Poly ADP-ribose polymerase
PDAC: Pancreatic ductal adenocarcinoma
PDLIM5: PDZ and LIM domain protein 5
PKC-ε: Protein kinase C ε
PKM2: M2 isoform of pyruvate kinase
PTBP1: Polypyrimidine tract binding protein 1
RBPs: RNA-binding proteins
RCC: Renal clear cell carcinoma
RTKs: Receptor tyrosine kinases
SCLC: Small cell lung cancer
SRC: Sarcoma viral oncogene homolog
TKIs: Tyrosine kinase inhibitors
TME: Tumor microenvironment
TMZ: Temozolomide
U2AF65: U2 small nuclear RNA auxiliary factor 65
UPR: Unfolded protein response
USP4: Ubiquitin-specific protease 4
VRACs: Volume-regulated anion channels
XIAP: X-linked inhibitor of apoptosis protein
ZEB1: Zinc-finger E-box binding homeobox 1
